# Meeting Highlights: 15^th^ International Mouse Genome Conference

**DOI:** 10.1002/cfg.127

**Published:** 2001-12

**Authors:** Jo Wixon

**Affiliations:** Bioinformatics Division HGMP-RC Hinxton, Cambridge CB10 1SA UK


**Comparative and Functional Genomics**
*Comp Funct Genom* 2001; **2**: 384–390.
**DOI:** 10.1002/cfg.127

**Meeting Highlights**

### 15th International Mouse Genome Conference

### EICC Edinburgh, Scotland, 21st–24th October 2001

Jo Wixon, Managing Editor
*Bioinformatics Division, HGMP-RC, Hinxton, Cambridge CB10 1SA, UK*

Published online:
20 November 2001

After the official opening of the meeting by **Ian Jackson** (**MRC Human Genetics Unit, UK**), **John McPherson** (**Washington University, USA**) gave his plenary lecture **Mapping and sequencing the mouse genome**. He started with a report on the status of 'the other genome' (human), stating that they now have ~2.9 Gb of sequence, 1.75 Gb of which is 'finished' and 99% of which is aligned to the chromosomes. Chromosomes 22, 21, 20 and Y are complete; chromosomes 6, 7 and 14 are nearing completion. In spring, the mouse genome sequencing consortium achieved their initial aim of 2–3X coverage, they are now doing more shotgun sequencing with the aim of reaching 5–6X coverage. This data will be better for assembly and regulatory element discovery purposes. They are using a combination of whole genome and BAC shotgun data, but have not as yet decided on the optimal ratio. They anticipate being able to increase the amount of machine time spent on mouse as the human genome is completed. Their BAC fingerprinting is done using highly controlled, reproducible gels; markers are then used to build the map up from the binning data provided by the FPC program. The contigs are then mapped onto the mouse radiation hybrid map and the human draft, which has also helped with gap length estimation. The map currently consists of 300 000 clones in >700 contigs and should be finished by the end of this year. There are currently two BAC maps, one made at Washington University (**http://genome.wustl.edu/gsc/mouse**) and one at the Sanger Centre. The mouse genome data is benefiting from annotation and display right from the start, unlike the human genome data (**http://mouse.ensembl.org**). Ensembl mouse uses the Sanger map and includes comparisons with the human data. Work is underway to link the mouse and human Ensembl resources to allow users to move between the two genomes. They are aiming for >99% coverage by early 2003 and a complete draft by 2005. There is also a SNP discovery project using a selection of strains, which it is hoped will identify 50 000 SNPs.

### Genome sequencing and comparative analysis

**Kerstin Lindblad Toh** (**Whitehead Institute, USA**) spoke about the **status of the mouse genome sequence**. At the moment the data stands between 2.75 and 3 Gb, which equates to 2.7X coverage. The sequence has been assembled into 650 000 contigs with an average length of 4.8 kb, 87% of these are anchored onto the BAC map. By February or March they hope to have 40 million reads and ~90–95% of the data anchored onto the map. The Homology Group at the Whitehead Institute have been looking at sequence conservation, using a selection of programs including GenScan, GenomeScan and TwinScan. Using TwinScan they think that they will find between 400 and 5000 new human genes. Of the 3% of sequence which is conserved ~half is coding and half is non-coding. The non-coding matches are typically 150–170 bp long and seem not very different from the coding matches. Many of them are within 1 kb upstream of genes and some are in introns. They have managed to cluster some of them and plan to further investigate the clusters.

**Shaying Zhao** (**TIGR, USA**) described the work at TIGR on their **mouse and rat BAC end sequencing projects**. For mouse they have two clone

Copyright © 2001 John Wiley & Sons, Ltd.

resources, with different average insert lengths (200 kb and 160 kb). They have sequenced over 400 000 ends from ~250 000 clones from the two collections. Their average read length is ~500 bp and they have sequenced both ends of ~190 000 clones. These have been assembled into contigs, and mapping the contigs onto the human draft genome has shown that they have a good spread of coverage. The rat project also uses two resources of BACs, this project is less complete and is still ongoing. So far they have sequenced at least one end of 47 000 clones from one resource and have 36 000 paired ends. The goal is to generate paired ends from 200 000 rat BACs in one year.

**Muriel Davisson** (**Jackson Laboratory, USA**) discussed their **comparison of human chromosome 21 with mouse chromosomes 16 and 17**. 60–70% of human chromosome 21 (from the centromere down) is conserved in mouse on distal chromosome 16, the remainder (working towards the telomere) is conserved in mouse on chromosomes 17 and 10. This group is interested in the region associated with Down syndrome; a mouse Down syndrome trisomy model that they have developed has a small extra chromosome with some of chromosome 16 and some of chromosome 17. They are sequencing these regions and have assigned many new genes to the two chromosomes. They have also identified the genes on human chromosome 21 which delineate the evolutionary breakpoints for the mouse chromosomes. In comparing human chromosome 21 with mouse 16, they have seen that of 91 orthologues, only one differs in gene order. There appear to be a few 'human specific' genes but these could lie in gaps in the mouse data. There are also a few apparently mouse specific genes, but they are not sure that these are real. Another interesting feature the comparison has uncovered is a conserved gene desert.

**Anne-Marie Mallon** (**MRC UK Mouse Genome Centre**) spoke about the **UK mouse sequencing programme** (**http://mrcseq.har.mrc.ac.uk/**). This is a collaborative effort, the mapping is shared between the MRC Human Genetics Unit, Imperial College and the MRC UK Mouse Genome Centre, and the sequencing is shared by the Wellcome Trust Sanger Institute and the MRC UK HGMP-RC. The team have chosen four targeted regions for study, which match ongoing UK research efforts, including three major target regions of the MRC ENU mutagenesis programme. They are still inviting applications for other regions to cover, until December. The regions covered so far are the WAGR-homologous region on chromosome 2, the region around the *Tyrp1* (brown) locus on chromosome 4, the Del(13) Svea36H (Del36H) region of chromosome 13 and the *Dmd* to *Ar* region of chromosome X. The mapping stage is nearing completion now and they have produced about half of the sequence. They are using Ensembl mouse to identify genes and have already refined the location of several genes. They are integrating the annotation from human and mouse Ensembl, and using *Fugu rubripes* data to identify upstream regions.

**Jim Thomas** (**NHGRI, USA**) presented the **large-scale vertebrate comparative genomics project** of the National Human Genome Research Institute and the NIH Intramural Sequencing Center. They have chosen 11 vertebrate species (based on the availability of BAC resources) for the project: chimp, baboon, pig, cow, cat, dog, mouse, rat, chicken, zebrafish and *Fugu*. They have taken a targeted approach, picking six regions of interest on human chromosome 7. For this they design evenly spaced probes against areas that are highly conserved in human and mouse. Using these probes they make BAC maps of each region of interest in each species and then select orthologous clones for sequencing. They use Pipmaker for alignments and graphical representations of data from three genomes at a time. Ultimately they want to compare all 12 together and are working on a MultiPipmaker tool. More closely related species (such as cat and dog and cow and pig) are better at corroborating matches, whereas less related species show different patterns of conservation. They plan to extend the study to more regions and to add more species (depending on the availability of BAC resources).

In his plenary lecture, **Gene Myers** (**Celera, USA**) spoke about **the Celera whole genome assembly for the mouse**. As at February 2000, they have 5.3X coverage. They have 11.25 million paired end reads (although some of these are not true, above 90% are true in each of their three resources). Their assembler philosophy is to detect and set aside repeats, and to first take advantage of the paired ends, i.e. they tackle the high confidence steps first. Their tool looks for >40 bp overlaps, allowing only 6% mismatch, and then constructs overlap consistent sub-assemblies. Repeat induced errors amongst these assemblies can be identified and discarded, since they are over-represented, often as much as 15–20X deep, as opposed to the expected 5X. The paired end data is now used to define

Copyright © 2001 John Wiley & Sons, Ltd. 

neighbours amongst the good assemblies, or 'unitigs', resulting in scaffolds. They require two or more paired ends joining two unitigs before they are assigned as a scaffold (Figure 1A). The paired end data also yields gap size estimates and is used to help close gaps. This is done by looking for reads pointing towards a gap and using their mate pairs (paired ends) to try to fill in the gap (Figure 1B). Next an STS map is used as an external reference to check the veracity of the map. They remain convinced that a 5X mammalian genome can provide an adequate substrate for annotation. They tried shredding the Celera and HGP data to make a new assembly, but the lower quality of the HGP data caused higher levels of uncertainty in the assemblies. However, using the 1.7X HGP mouse data with their human data did improve the assembly, the span remained essentially the same, but as less scaffolds, with less gaps. 80% of their mouse data is in scaffolds >10 Mb in size, with 97% in scaffolds >1 Mb. 50% of the gaps are <100 bp and there are 72 conflicts with the genetic marker order so far. Comparing human chromosome 21 and mouse chromosome 16, the mouse genome appears to be 10–15% smaller than human. They have annotated their mouse assembly using the same tools developed for the human data, which predict roughly similar numbers of genes for mouse compared to human, at ~30 500 very high confidence predictions rising to ~40 500 at a lower confidence level. They plan to use RIKEN clones for alternative splicing data.

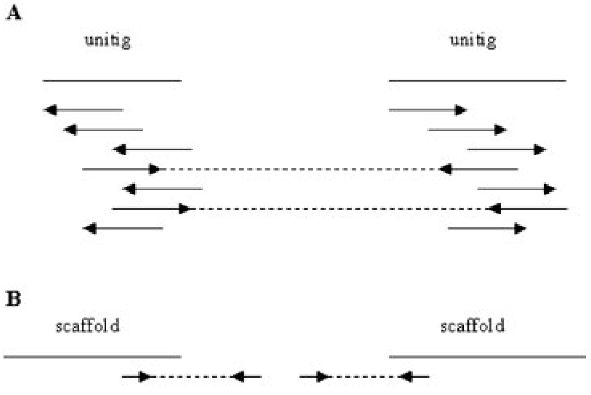

**Figure 1.** Stages in the Celera scaffold assembly process. A: If two or more paired ends can be used to define two contigs as neighbours they become a scaffold. B: The paired end reads of reads pointing out into gaps are used to help close gaps. Arrows denote individual reads and dashed lines identify paired ends, or mate pairs

### Functional genomics

**Tim Wiltshire (GNF, USA)** presented the results of **a large-scale analysis of the mouse transcriptome**. They have used high-density oligonucleotide arrays to study the expression of 8882 human genes in 46 different tissues and 6139 mouse genes in 45 tissues. The results for 1800 genes were verified by RT-PCR and other genes were checked by Northern blot and *in situ* analyses. 90% of the genes gave a signal in at least one tissue. They have constructed a gene expression database (**http://expression.gnf.org**, but it is not available yet) and have also performed clustering of genes by their tissue expression patterns. This has led to the identification of upstream regulatory elements and interesting observations on the tissue restriction patterns of gene families. They have defined 799 putative orthologues between human and mouse, about half of which show a correlation coefficient of over 0.6 for their expression. They also observed that some tissues showed a better overall correlation between human and mouse.

**Lee Smith (MRC Mammalian Genetics Unit)** reported on **a microarray screen for genes involved in mammalian sexual development**. They are using the array to compare male and female embryo samples; they do four replicates, two with one colour orientation and two with the reverse colour orientation. All genes giving interesting results are checked by *in situ* analysis to validate the result and allow observation of timing and cell specificity of expression. They have detected know sexually dimorphic genes and identified ~350 new candidates. However, the technique requires too much sample (from tiny embryonic organs) for complex studies such as timecourses to be really feasible, so they have developed a modified amplification technique to reduce the quantity of starting material required. Looking at their overall expression plots, they do see major differences in each tissue at the times expected from other studies, and so they feel that the array data is matching the known biology. They are now looking at mutants of known and candidate sexually dimorphic genes.

**Harukazu Suzuki (RIKEN, Japan)** presented **an analysis of protein-protein interactions detected using the RIKEN full-length cDNAs**. They have used a PCR-based *in vivo* screen to detect interactions. Their 2-hybrid approach is performed in microtitre plates, giving high-throughput, and each protein is used as bait and prey giving better confidence in the

Copyright © 2001 John Wiley & Sons, Ltd.

results. In testing using the Wnt pathway, all but one of the known interactions were detected. 6000 cDNAs were chosen for the assay (around 2% of baits had to be discarded), which detected 505 interaction pairs. They have written a program, called PPI network viewer to handle their data. Proteins are denoted by circles, joined by arrows representing the interactions, the thickness of each arrow being used to denote the strength of the interaction. The viewer has options to restrict the level of the network, by the interaction strength or by the length of links between proteins. The viewer has links out to expression profiles and mapping data. They expected to see a correlation between the expression profiles of genes they had identified as interaction pairs, and there was a significant but not pronounced trend towards this in their data. However, looking also at data from DIP, they found that the effect is not enough to allow prediction of interaction pairs from expression data.

**William Stanford** (**University of Toronto, Canada**) spoke about **a large-scale gene trap mutagenesis-based expression and genotype screen**. The traditional gene trap system works by insertion of a construct with a splice acceptor site followed by a promoter-less reporter gene. If the construct inserts into an intron, the reporter gene shows the expression pattern of the trapped gene. Vectors that use splice sites need to trap a downstream poly A tail signal to work, so they have included a poly A trap vector, designed to get around this problem, in their selection of 6 vectors. They have created >5000 trap clones, >4500 have been screened and 3000 have been analysed. They now have funding to generate sequence tags for all of them, to identify the position of the insertions. A database is being created which will allow users to search by expression pattern, sequence, and phenotype data from their ongoing screens (**http://www.cmhd.ca**).

**Using puffer fish DNA sequence to analyse mammalian genomes** was the title of **Jean Weissenbach's** (**Genoscope, France**) plenary lecture. They have been shotgun sequencing the genome of *Tetraodon nigroviridis*, a smaller relative of *Fugu rubripes*, which is easier to maintain. They are using a combination of BAC fingerprinting, *in situ* hybridisation and STS mapping to build their map. They are also comparing their data to the human sequence and other genomes, to identify evolutionarily conserved regions, which they call 'ecores'. These are particularly useful for identifying human exons and will contribute to a definition of a vertebrate gene set. Using the initial chromosome 22 data, they estimated that their e-cores detected 66.8% of exons from known genes, 55.3% of exons from related genes and 14.8% of exons from predicted genes. Taking the data from the reannotation of chromosome 22, their scores improve to 70%, 60% and 20% respectively. As of June 2001 they have almost 3X coverage, assembly of the sequences is still underway, but comparison of the new sequences with human data has given them more ecores. However, the number of ecores per gene is less in these new sequences, which he suggests could indicate that they are now finding the less well-known genes. The Whitehead Institute has independently been sequencing the same genome, they have also reached around 3X coverage, and the two groups have agreed to combine their data. As yet, they have made no comparisons to the *Fugu rubripes* data.

In his plenary lecture, **Tim Hubbard** (**Wellcome Trust Sanger Institute, UK**) discussed **the annotation of vertebrate genome sequences**. He spoke about Ensembl mouse (**http://mouse.ensembl.org**), which is very like the human Ensembl resource (**http://www.ensembl.org**). It is possible to search against the mapped clones, the Sanger assembly and the Whitehead assembly, and the display even includes the BAC fingerprinting assembly. 71.2% of the Whitehead assembly has been mapped back onto the genome, giving ordering and orientation data, which will lead to a mouse 'Golden Path', but this is not yet available on Ensembl mouse. Their gene annotation group is currently working on human chromosomes 6 and 13, and a gene identification group will be sequencing full-length mRNAs across the whole human genome. Taking their annotation further includes searching for promoter regions and he presented a tool called Eponine which has been written for this purpose (**http://servlet.sanger.ac.uk:8080/eponine/**). One feature of Ensembl that he is keen to promote is the use of a distributed annotation system (DAS, **http://biodas.org**, **http://www.ensembl.org/das**) which allows users to coordinate synchronisation between distributed resources and serves your data to other resources using XML.

### Informatics

**Deanna Church** (**NCBI, USA**) gave an overview of the **resources for mouse at the NCBI**. At the level of sequence annotation, they are providing data

Copyright © 2001 John Wiley & Sons, Ltd. 

such as known genes, EST clusters, STSs, SNPs, GenomeScan models etc. and they are striving to integrate nomenclature, disease genes, non-sequence-based maps. They offer multiple query options, including BLAST searches, text queries and location-based queries. They offer a range of maps, the MGI composite map, and the Whitehead Institute genetic, YAC and radiation hybrid maps can be displayed using their Map Viewer tool, the fingerprinting and comparative maps are not yet on Map Viewer. Their Refseq resource holds 7459 mRNAs and 484 genomic contigs, in addition they have a huge amount of raw data. Users can compare human and mouse data and also compare the NCBI and UCSC human assemblies against mouse data to try to reduce conflicts in the maps.

**Carol Bult** (**The Jackson Laboratory, USA**) discussed their work on **integrating computational and human-curated annotations for the mouse genome**. They have manually curated sets of gene sequences, which could be used to aid computational annotation, and for validation of the annotation. She described their infrastructure for combining these datasets. The first stage is a rapid, automated binning process, based on alignment data (Figure 2). This separates the entries into genes not yet represented in one of the resources, unique genes represented in both resources, and more complex cases, which will need manual annotation. Before the inclusion of the FANTOM data, their Mouse Genome Informatics group (MGI) had ~12 000 genes, after adding the FANTOM data, they had ~24 000 genes and they now have ~40 000 genes (alternate splice forms have been merged to achieve this number, but there may be other reasons for the apparent redundancy). They are also incorporating the RIKEN data into their analysis, to aid gene detection using the Clone Curator tool from Berkeley. ~50% of the MGI genes can be associated with an electronic transcript in the TIGR resource and they are now trying to link these directly. They are also collaborating with NCBI and Ensembl for the genome assembly and data representation and they will be using the VISTA tool to provide graphical representations of human-mouse comparisons.

**Monica Justice** described how to achieve **large-scale isolation and rapid mapping of recessive mutations using a mouse balancer chromosome** in her plenary lecture. Ethylnitrosourea (ENU) causes point mutations (by alkylating bases in DNA),

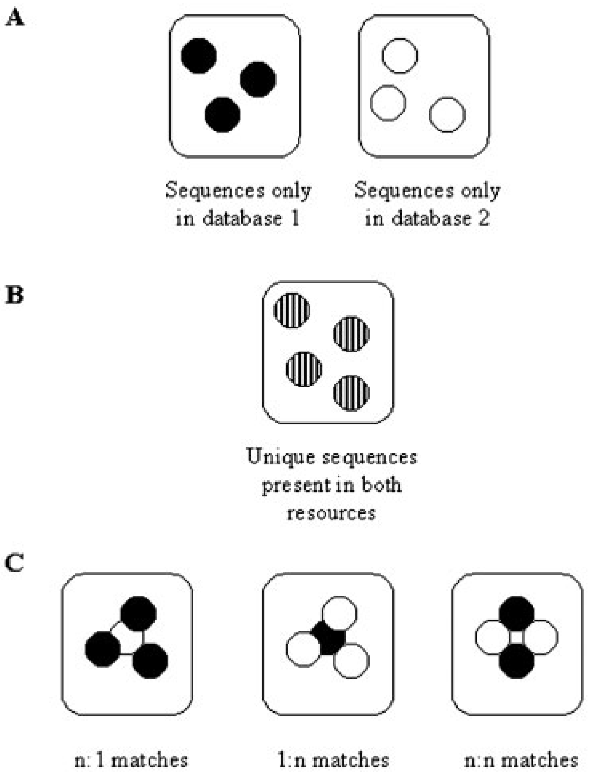

**Figure 2.** The bins used to sort results from comparing two resources. A: Genes absent from one or other resource B: Unique genes represented in both resources C: More complex situations that are flagged for manual annotation which can model the point mutations that cause human disease. To generate genome-wide mutations, researchers treat the male germline with ENU, mate these mice and then screen for mutations. Using the Cre-*lox*P system it is possible to make inversions, deletions or duplications of particular regions of the genome, whilst inserting a reporter gene to visibly tag mutant mice. Using this strategy with Embryonic Stem (ES) cells, they have produced a series of deletions and inversions on chromosome 11 tagged with coat colour markers (**http://www.mouse-genome.bcm.tmc.edu**). These can act as balancer chromosomes in matings with ENU mutagenised mice. Progeny with a mutated chromosome 11 and the balancer are then mated with mice carrying the balancer. Dominant mutations are picked up in generation one, recessives in generation three, which is faster than traditional approaches. If the mutation is lethal, this approach still yields carriers. They look for visible phenotypes and perform fertility screens and biochemical tests on

Copyright © 2001 John Wiley & Sons, Ltd. 

blood and urine of the progeny. So far, they have identified >155 recessive mutations, of the 63 they have mapped so far, 62 are on chromosome 11, as expected, none are allelic, indicating that they are far from saturation. Only four show non-Mendelian inheritance. Of the 39 genetic lethals, 23 die at or before birth, two die before weaning, six show semi-viability and eight are sterile. ~30 of these are previously described visible phenotypes from other genome-wide studies. The lethal mutations can be good models for haematopoesis and vasculogenesis, they use the yolk sac as a reporter of blood vessel development. 11 of the 21 lethal mutations they have studied in this way die at stage E9.5–12.5, when the vasculature is developing. One has no yolk sac vasculature at all.

### Mutagenesis

**Karen Steel (MRC Institute of Hearing Research, UK)** reported on **mouse models for hearing and balance defects** obtained from the European mutagenesis programmes. First she explained that there are many forms of human deafness, many of which have no models. In a three year screening project, they have tested 53 000 mice, uncovering >50 new mutants. These were produced 17 of these have been mapped to a chromosome and six have been identified. 8 of the mutants show a novel phenotype. Of the three middle ear defects, one is a mutation in *Tcfap2a* on mouse chromosome 13. There are two stereocilia defects and three organ of corti defects (some of these overlap with other phenotypes). Of the inner ear defects (all of which have truncated semicircular canals), two are mutations in the *Jag1* gene, and eight are mutations in a second gene, on chromosome 4. There are also several progressive hearing loss phenotypes.

**Heinrich Flaswinkel (Technical University of Munich, Germany)** spoke about **phenotypic analysis and chromosomal mapping of mouse mutants with immunological defects** caused by ENU mutagenesis. Their dominant screen involves mating mutagenised C3H males with females from the same strain. They use an array of ELISA assays to test the progeny for levels of a wide range of immune proteins, and FACS analysis to assess the numbers of each type of immune cells. They have so far analysed ~9000 mice obtaining 54 confirmed mutants. Examples include a mutant with a four fold reduction in IgG2a, a mutant with reduced numbers of CD8+ cells, a mutant with elevated IgM levels, and a mutant whose peripheral CD8+ T-cells are also CD4+. They have also performed a recessive screen, in which they have tested 1390 mice (this is complicated by high rates of infertility). This screen has identified more severe mutations, including one with near complete loss of complement receptor 2 on its B-cells and one with no T-cells at all.

**John Schimenti (The Jackson Laboratory, USA)** described their work on **mouse infertility and genome instability mutants**. They have used ENU mutagenesis of males, and of ES cells. Their screens have identified mutants in all stages of spermatogenesis, including meiotic arrests, and mutants with crucial cell types missing. They also have some oogenesis mutants. During meiosis, synapses are formed, proteins accumulate on the chromosome pairs, holding them together (synaptonemal complex), then DS DNA breaks are formed and fixed, forming crossovers (recombination). One of their arrest mutants makes the breaks, but appears to lack a checkpoint, such that the breaks are not fixed, causing the arrest. Another shows no pairing of chromosomes, which leads to a chaotic organisation of the chromosomes during division. They have also used a micronucleus assay to find mutants showing genome instability.

**Jay Vivian (University of North Carolina, USA)** reported on **a genotype-based screen in mouse ES cells for ENU-induced mutations** in the SMAD2 locus. This method allows researchers to identify unmarked mutations, in ENU-treated ES cells, in their gene of interest. They treat ES cells with a regulated dose of ENU. Surviving cells are plated in duplicate, one plate is used for D-HPLC mutation detection, the other is stored as a frozen library, for the subsequent production of chimeric mice. The SMAD 2 and 4 genes were chosen to test the approach, as they are expressed in ES cells and not too big to screen for mutations. They are of interest as they potentially mediate development and tumour progression. The SMAD 2 knockout is lethal at E6.5-7.5 and no proper embryo is formed, limiting further study. Using this approach, they have obtained a range of mutants, which they hope will allow them to learn more about the phenotype, one in particular does make a badly deformed embryo, allowing further study of the role of the gene.

**Emma Coghill (MRC Mammalian Genetics Unit, UK)** presented **a gene-driven approach to the identification of ENU mutants** in the connexin 26

Copyright © 2001 John Wiley & Sons, Ltd.

gene. This approach benefits from having parallel DNA and sperm archives from ENU mutagenised mice. The DNA archive was screened for mutations in the connexin 26 gene (*Gjb2*, a gap junction protein linked to hearing loss) and for those samples showing mutations, the mouse was 'resurrected' from the sperm archive. In the *Gjb2* knockout, heterozygotes are normal, but the homozygotes are lethal hindering further analysis, this technique generates different types of mutants that might allow further study. They used Transgenomic Wave™ (a mismatch heteroduplex detection system) to identify mutants in Gjb2 and then sequenced the gene to see what each mutation was. They have been able to do limited pooling of the samples to be analysed, at most they recommend four into one. They found one mouse carrying a STOP mutation and used the sperm resource to regain the mouse. From a cross between its progeny, they were unable to obtain any homozygotes, but have 37 heterozygotes. They are now searching for other alleles of this gene and extending their screen to other cochlear-expressed genes.

**Allan Balmain (UCSF, USA)** discussed approaches for **identifying mouse and human cancer modifier genes using mouse models**. Modifier genes are host genes that have an effect or tumour progression. In the case of familial cancer, the host background has ~5% effect (only one fifth of the breast cancer risk in BRCA families is due to BRCA. High penetrance genes have a high relative risk, but are rare, low penetrance (or modifier) genes can be very common. However, they are involved in genetic interactions and are very difficult to map. There could be as many as tens to hundreds of these modifier genes. So, how do we find them? SNP scans would need ~500 000 SNPs to be genotyped in 1000 cases and controls, which would cost billions of pounds, so alternative approaches are needed. His group have a mouse model of chemically induced skin cancer. Comparing *Mus spretus* and *Mus musculus* they saw that *Mus spretus* was resistant to the chemical treatment, and making crosses gave increased susceptibility. They are now looking for the underlying genes. Many tumour susceptibility loci have already been mapped in the mouse, including several for skin cancer, there are >20 in just one *spretus musculus* cross. They try to narrow down regions by subdividing phenotypes, typically they are starting with 10–20 cM regions identified by linkage analysis. Making congenic mice and crossing then tracking down the phenotype is one approach, but this is very slow and they are exploiting alternatives. They are looking at outbred mice, making many different crosses and identifying polymorphisms that are only present in mice that show linkage. An interspecific backcross of these mice with the inbred parent and mating the progeny of that mating back to the inbred parent again can separate different outbred alleles. This approach can get the region down to ~1 cM, at which point they look for candidate genes in the region and sequence those genes in (human) individuals to find SNPs to test for association, which then identifies human candidate genes.

---

The Meeting Highlights of Comparative and Functional Genomics aim to present a commentary on the topical issues in genomics studies presented at a conference. The Meetings highlights are invited and each represents a personal critical analysis of the current reports and aim at providing implications for future genomics studies.

Copyright © 2001 John Wiley & Sons, Ltd. 

